# Violence against physicians and nurses in a hospital: How does it happen? A mixed-methods study

**DOI:** 10.1186/s13584-017-0183-y

**Published:** 2017-10-31

**Authors:** Sigal Shafran-Tikva, David Chinitz, Zvi Stern, Paula Feder-Bubis

**Affiliations:** 1 Hadassah University Medical Center, P.O.B 12109, Kiryat Hadassah, 12000 Jerusalem, Israel; 2 Health Policy and Management in the School of Public Health, Hebrew University-Hadassah Israel, Jerusalem, Israel; 3 Hadassah Mt Scopus Hospital in Jerusalem, Jerusalem, Israel; 4 Department of Health Systems Management, School of Public Health, Faculty of Health Sciences and Guilford Glazer Faculty of Business and Management, Ben-Gurion University of the Negev, P.O.B. 653, Beer-Sheva, 8410501 Israel

**Keywords:** Coping (with violence), Hospital conditions, Patient behavior, Prevention (of violence), Roles and responsibilities, Staff behavior, Violence, Waiting times

## Abstract

**Background:**

Violence against medical personnel is unexpected in hospitals which are devoted to healing, and yet, it is frequent and of concern in the health system. Little is known about the factors that lead to hospital violence, and even less is known about the interactions among these factors.

The aim of the study was to identify and describe the perceptions of staff and patients regarding the factors that lead to violence on the part of patients and those accompanying them.

**Methods:**

A mixed-methods study in a large, general, university tertiary hospital. A self-administered survey yielding 678 completed questionnaires, comprising 34% nurses and 66% physicians (93% response rate). Eighteen in-depth interviews were conducted separately with both victims and perpetrators of violent episodes, and four focus-groups (*N* = 20) were undertaken separately with physicians, staff nurses, head-nurses, and security personnel.

**Results:**

Violence erupts as a result of interacting factors encompassing staff behavior, patient behavior, hospital setting, professional roles, and waiting times. Patients and staff reported similar perceptions and emotions regarding the episodes of violence in which they were involved. Of 4,047 statements elicited in the staff survey regarding the eruption of violence, 39% referred to staff behavior; 26 % to patient/visitor behavior; 17% to organizational conditions, and 10% to waiting times. In addition, 35% of the staff respondents reported that their own behavior contributed to the creation of the most severe violent episode in which they were involved, and 48% stated that staff behavior contributed to violent episodes. Half of the reasons stated by physicians and nurses for violence eruption were related to patient dissatisfaction with the quality of service, the degree of staff professionalism, or an unacceptable comment of a staff member. In addition, data from the focus groups pointed to lack of understanding of the hospital system on the part of patients, together with poor communication between patients and providers and expectations gaps.

**Conclusions:**

Our various and triangulated data sources show that staff and patients share conditions of overload, pressure, fatigue, and frustration. Staff also expressed lack of coping tools to prevent violence. Self-conscious awareness regarding potential interacting factors can be used to develop interventions aimed at prevention of and better coping with hospital violence for both health systems' users and providers.

## What is already known about the topic?

- Violence towards healthcare workers in hospitals is a widespread phenomenon
- Work overload, waiting times, and nurse-patient relations have a major impact on hospital violence

## What this paper adds

- Staff behavior plays a role in the creation of violence in hospitals.
- Violent incidents erupt due to the constellation of multiple factors: staff behavior, patient behavior, hospital conditions, waiting times, and undefined roles and responsibilities.
- Both sides, patients and staff, involved in a violent episode have feelings of fear, frustration, loss of control, pressure, overload.
- Involving security personnel in a violent event should be done judiciously, as it was reported contributing to the escalation of the situation.

## Background

Both a literature survey and daily news accounts point to the fact that violence is frequent throughout the world. It is expressed every day in public spheres such as the education system, the health system, the legal system as well as others. Violence in the healthcare system can mirror violence in society in general. But violence seems to be almost counterintuitive to socially held expectations regarding hospitals, sites that symbolize security, care, compassion, and life-saving. It is difficult to accept that violent incidents take place frequently in hospitals and in some cases of physical violence can even turn a hospital into a battleground [1].

Violence against hospital medical staff manifested in disparagement, insults, threats, and physical harm is a widespread and worrying phenomenon. Studies from around the world illustrate the extent of this phenomenon that crosses cultures and borders [2–8]. For example, in the UK, a number of studies demonstrated the high prevalence of violence phenomena in medical wards as well as among different professions [9, 10]. In China, a country with the lowest murder rate in the world, 29 murders in hospitals were reported between 2001 and 2011 [11], certainly a high number in absolute terms (data on total hospital contacts was unavailable). In 25 Israeli emergency rooms, 87% of the staff reported having suffered violence directed at them by patients or their visitors during the past year [12].

Despite the significance of understanding violence in the context of hospitals, most studies of health system violence limited themselves to the measurement of its extent, and mainly focused on emergency and psychiatry wards. Other studies focused on violence between staff members [13–15], and on violence from staff to patients [16–18]. Only a few studies have addressed the causes of violence, and it seems that, to date, three main clusters of factors have been found to lead to the development of violent episodes: environmental factors, patient-related factors, and factors related to communication between nurses and patients [19]. Among the first, lengthy waiting times and conditions such as crowded space, contribute to violence occurrence. Beyond those, other studies suggested that in addition to relatively simply measurable factors such as work overload and waiting times, nurse-patient relations have a profound influence on hospital violence [20–22]. Gaps between staff and patient perceptions were discovered regarding the reasons for violent episodes and the manner in which staff coped with aggression aimed at them [23] Moreover, as revealed in a companion paper violence assumes different forms and occurs with varying frequencies across different departments, suggesting that perceptions of those involved in violent episodes need to studied more closely [24].

Violence in hospitals has several negative impacts: physical and mental harm to the attacked person (clinical and administrative staff), who suffers from violence sequels in the short and long terms [25–28], high costs for the organization, and possible reduction in the quality of care received by violent patients [29]. Thus, understanding the overall picture of violent events is critical for designing tailored intervention programs for coping with this growing problem.

Given the magnitude of violence between hospital teams and patients (along with their accompanying companions), and the gaps in the perception of its root causes, there is a need to examine in-depth the causes, processes and factors that contribute to the phenomenon of violence from the perspective of all involved. This study was designed to inquire into the constellation of factors and processes that contribute to create a violent episode between patients and staff in a hospital setting. The perspectives of all involved - nurses, physicians, patients and their companions, and security personnel – were included. In the next section we describe the methodology of the study. This is followed by the presentation of the categories emerging from the data analysis, showing that episodes of violence arise and unfold due to multiple interacting factors. After discussing study limitations, we conclude with implications for policy and future research.

## Methods

### Study design

A case-study methodology was chosen [30] to holistically appraise the contingencies leading to violent events in hospitals. Due to the complexities of the phenomenon, we decided to undertake the study in a specific setting whose members clearly the respondents share organizational and cultural contexts. The case-study was conducted in a large, urban, tertiary university medical center. It comprises almost 700 beds, and employs approximately 5000 persons, including 750 physicians and around 1000 nurses. The hospital offers many advanced medical services, community health programs, and outpatient clinics. During an average day, an estimated 30,000 persons visit the hospital.

In order to inquire into what would be unequivocally defined by both sides as a violent episode, we focused on events that involved the security personnel of the hospital. However, in order to interview patients who were involved in violent episodes, as described below, we were limited to events that were documented because security personnel were summoned. The advantages of this approach is that it enables the most complete assessment of a violent episode. We are aware that security guards are not called in all cases and that some hospital violence may go unreported (and thus, not researched). Nonetheless, episodes of violence that were not reported and impact on overall hospital atmosphere and quality of care are reflected in the data, as we also know from our companion research [24].

The case-study included both qualitative and quantitative components, and comprised in-depth interviews with dyads of victims and perpetrators of violence, four focus-group discussions, and a self-administered survey. This multi-pronged approach enhanced the trustworthiness of the study findings, supported by triangulation of evidence.

### The qualitative component

Qualitative data collection was carried out during May–November 2010, and included a. focus-groups discussions, b. in-depth interviews, and c. open-ended questions, included in the quantitative survey.

**a. Four focus-groups** were undertaken with five head-nurses from ambulatory clinics; five staff nurses (who work three or more times per week only in the emergency ward); five physicians (specialists and residents) from five clinical specialties; and five full-time security personnel, first responders to violence incidents Focus groups participants’ demographic data is exhibited in Table 1. The objective of the focus-groups was to obtain perspectives of different groups of hospital employees.

**Table 1:** Focus groups participant’s characteristics

Participants	Gender		Seniority		Participants’ age range
	F	M			
Head nurses	5		≤5 yrs	≥6 yrs	43–62
Physicians	1	4	≤5 yrs	≥6 yrs	36–48
Staff nurses	4	1	≤5 yrs	≥6 yrs	27–51
Security personnel	4	1	4 months	7 years	22–43

In a telephone call, participants were invited to participate in a focus-group study about violence against physicians and nurses. All of them agreed almost immediately, emphasizing the importance of the issue. A reminder was sent via email, 4 days before the scheduled meeting.

Participants typically arrived on time for the meeting and in general, the atmosphere in all groups was relatively informal. Each discussion lasted about one and a half hours. There was a sense of openness and willingness to talk about a variety of aspects related to violence. For example, at the outset of a focus-group, a resident physician stated: “I wanted to say a few things which I believe are really important, and that’s why I came here.” Topics discussed related to the genesis of violent events, staff and patient behavior before and during episodes of violence, and perceptions regarding other factors impacting on the violent occurrence. The interaction of participants in the focus-groups produced varied and meaningful insights for understanding hospital violence between staff and patients in a comprehensive way.

**b. In–depth interviews:** Every morning, in the spring of 2010, a researcher (SST) retrieved information from the security department database about reported violent episodes that took place in the hospital, aiming to interview the involved. We planned this qualitative component so as to include patients’ or their companions’ voice. We did so until theoretical saturation was reached. Eighteen semi-structured, in-depth interviews were carried out with staff who were attacked and with perpetrators, regarding the same incident. Violent episodes referred to in the interviews were those in which security personnel were called to intervene. The objective of these pairs of interviews was to understand, with detail, different angles of the development and contingencies of specific violent episodes. Each interview took place within 72 h of the event in question, separately with the staff member and the attacker. Interviews lasted between 40 and 60 min, and took place according to the preference of the participant – in the interviewee’s office, by phone, next to the patient’s bed, in the ward staff-room. Privacy and lack of interference were assured. The patients were heterogonous, although for reasons of confidentiality we did not ask collect socio-demographic information from the interviewees.

Interview topics included the description of the specific violent event, the roles played by all involved, its contributing factors, and emotions that arose during the episode. Each interview began with the detailed description of the violent event, followed by the participant’s interpretation of it.

These interviews were intense and breathtaking. Participants were eager to tell their story and expected empathy (“it’s my mother, after all”) and, in the case of staff, solidarity (“you know what I’m talking about”). One gets the sense of the dense unfolding of events and the “little” factors and behaviors that can spark an incident.

Similar to the focus-group discussions, all interviews were tape-recorded, transcribed verbatim, and checked for accuracy.

**b.** Two open-ended questions aimed at eliciting the factors that contribute to violent episodes, linked to hospital staff and independent of hospital staff, were included in the quantitative survey in order to enable respondents to articulate and expand their personal views and/experiences.

### The quantitative component

A quantitative survey was carried out using questionnaires during May–October, 2010. Despite the long period since data was collected, no significant organizational changes have taken place regarding violence in this hospital; neither have changes taken place at the national level due to lack of changes in policy regarding violence. Questionnaires were filled out by a sample of physicians and nurses after obtaining approval from ward directors and head-nurses. The questionnaires were distributed during staff meetings of a variety of clinical divisions (surgery, oncology, intensive care, ambulatory services including day care, and emergency room). The questionnaire was completed by 230 (34%) physicians and 446 (66%) nurses. The overall response rate was 93%.

Besides providing demographic data, respondents answered questions about coping with the most severe case of violence directed at them, the degree of contributions of the respondent and other staff to the violent incident, and level of agreement with 36 statements regarding violence, measured on a seven-point Likert scale. A full analysis of these attitudinal data is beyond the scope of this paper. Here we draw only on those results relevant to the causes of violent episodes.

Before its distribution, the survey questionnaire was assessed by three physicians and five nurses from different wards and of different ethnic backgrounds. After their suggested corrections, the questionnaire was further appraised by 11 key members from different sectors of the hospital administration. The feedback received indicated that the questionnaire was comprehensive, friendly, clearly stated (except for a few proofing comments), and can be used to evaluate the degree of exposure of staff to violence and to identify the factors that contribute to the formation of violent events. (Table 2)

**Table 2:** Distribution of demographic and professional variables in the survey

Variable	Description	N (%)	Total (n)
Age (mean yrs.; sd)		11.2 ± 40.6	(100%) 641
Gender (n;%)	Male	(39.9%) 270	(100%) 677
	Female	(60.1%) 407	
Physicians’ roles (n;%)	Department Head	(3.4%) 23	(34%) 230
	Senior physician	(15.5%)104	
	Resident	(12.8%) 86	
	Intern	19 (2.8%)	
Nurses’ roles (n;%)	Head nurse	(4.9%) 33	(66%) 446
	Staff nurse	(55.7%) 375	
% Full time equivalent (FTE) ≥ 50% (n;%)	Nurses and physicians (total sample)	(98%) 635	(100%) 643
Professional seniority (mean yrs.; sd)	Nurses and physicians (total sample)	11.2 ± 14.3	641 (100%)
Departmental seniority (mean yrs.; sd)	Nurses and physicians (total sample)	8 ± 8.6	(100%) 646

Table 1 displays the characteristics of the survey respondents.

More than 50% of the respondents work in the surgery and internal medicine divisions. Approximately, 12% work in oncology; 11% in intensive care and 9% in ambulatory care services.

### Data analysis

#### The qualitative component

**Fig. 1:**
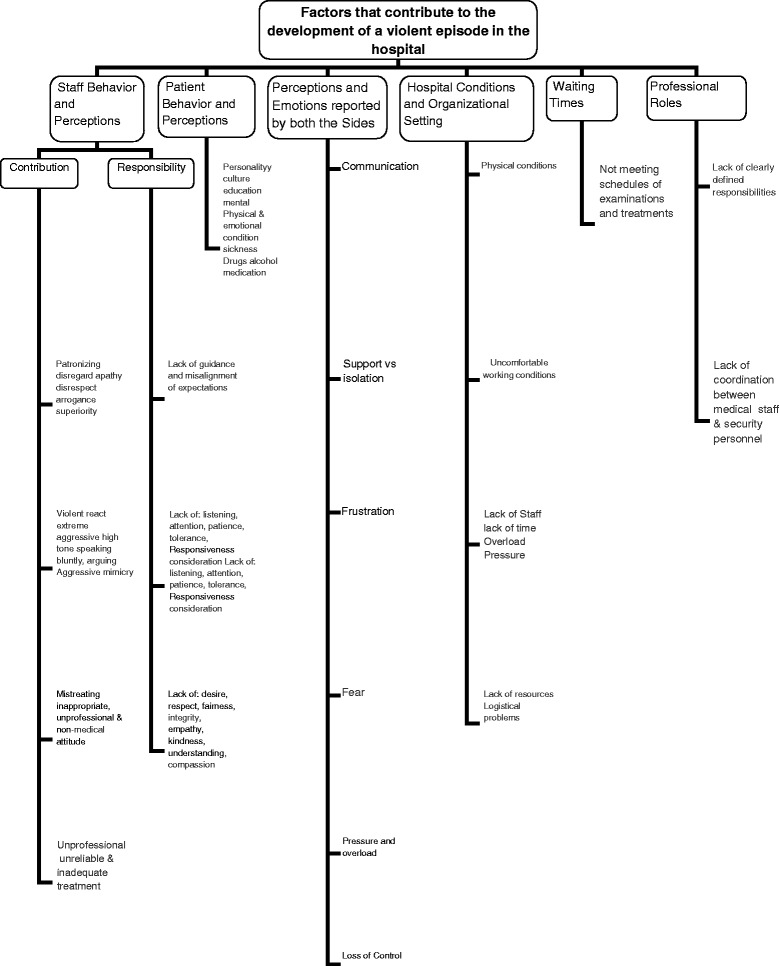
Array of factors contributing to the formation of violence (p. 22)

Qualitative data from focus-groups, interviews, and survey open-ended questions were analyzed using grounded theory. The transcriptions of focus-groups and in-depth interviews, together with the texts of the open-ended questions from the survey were re-read before being split into meaning units. Meaning units were then grouped into categories, that were labeled in-vivo, i.e. based on the actual words of the respondents. The categories were subsequently mapped and re-mapped, with the implied relationships among them, helping in their refinement into sub-categories. Further exploration of the relationships among the categories in light of the theoretical background and the initial research questions resulted in the mapping displayed in Fig. 1, in the findings section. Verbatim quotations of study participants illustrate study categories.

#### The quantitative component

The data collected were analyzed using SPSS version 12. All tests were two tailed and significance was determined at .05 confidence interval.

### Ethics, trustworthiness, and study validity

The investigator who collected the data (SST) is a senior nurse who has worked in both clinical and administration wards in the hospital. This enabled ready access to respondents and an understanding of “where they are talking from”. In order to ensure reflexibility, peer debriefing sessions with the other researchers, one of whom is a hospital director, provided a sounding-board for the interpretation and presentation of the data. Thick description was employed to focus the investigators’ attention on the data collection process in order to consciously assess the impact of the data collector’s closeness to the field of study. Confidentiality was ensured to all study participants, which encouraged their sincerity.

In order to obtain a better understanding of the organizational structure of the security department and the roles of security personnel, the principal researcher conducted an informal conversation with the head of hospital security, prior to the security staff focus-group discussion. In addition, in order to allow accurate analysis of the data collected from the security staff, one of the focus-group participants and the head of hospital security read the transcript of the discussion for feedback and corrections. In all cases, the text was approved without change.

Permission to conduct the research was obtained from the institutional review board of the hospital, as well as from its directorate. All participants received full disclosure of the purposes and process of the research. Their voluntary participation and its tape-recording was by consent.

## Results

In this section the combined analysis of the quantitative and qualitative findings will be presented according to the categories that arose from the data analysis.

Analysis of the focus-groups, interviews, and open-ended questions in the survey produced six main categories of factors that contribute to the development of a violent episode in the hospital: 1. Staff behavior; 2. Patient behavior; 3. Perceptions and emotions reported by both sides; 4. Hospital environment; 5. Waiting times; 6. Professional roles and responsibilities. It seems that it is *the constellation of these factors that matters*, that is, all or part of them might contribute to various extents and circumstances to different violent incidents (Fig. 1).

As a prelude to the presentation of these categories, it is worth noting the contexts and interactions that were often depicted as the incubators for episodes of hospital violence. What stands out is that these situations are typical of the process of care, such as:

- The transfer of a patient between wards in the middle of the night;
- A family member sitting on a clean bed (prepared for a prospective patient);
- A patient or family member request to be treated only by a Jewish physician;
- A family member asking for assistance in changing a colostomy bag;
- A staff member requesting guests to leave because visiting hours are over.

Even when the facts reported were similar, these interactions’ narratives and their meanings differed considerably between those attacked and the perpetrators. In particular, the language used describing the event was different in terms of slang, body language, tone of voice, and the content of the “dialogue”. It seems that neither side heard the other, and each explained and interpreted the event from their own point of view, justifying their own behavior, while attributing responsibility to the other side.

*“If she [a nurse] would only turn to me more politely … if she would just listen to me … she caused all this story … ” (Patient).*

These subtle differences in perception are laced through the categorized findings to which we now turn.

### Category 1. Waiting times

Waiting time as a factor contributing to violence came up in the analysis as a distinct category, beyond the factors that will be detailed in the following categories. Participants expressed “understanding” of violent behavior due to long waiting times.

*“You see how people wait four, five, six hours. I have to say, as someone who is part of the system, that it is very often not justified, absolutely, hand on my heart.” (Physician)*

*“What causes violence in my clinic is the long waiting time which is out of proportion.” (Head-nurse)*

In response to the open-ended questions in the survey, 384 (9.5%) of the statements elicited related to waiting times for examinations, treatments, physician consultations, and imaging. Most of the respondents linked waiting time to factors dependent on the staff (244, 63%), and the rest (140, 37%) suggested that waiting time is caused by other factors in the hospital setting. Seventy-five percent of the respondents stated that waiting time was an important contributor to violent events. But, when taken into account together with the additional finding that only 23% of the respondents felt that waiting time was excessive in cases of violence, it becomes clear that the notion of “waiting time” has to be unpacked and is also possibly subject to staff behavior. It seems that staff and patients perceive the length of waiting time differently, and staff need to be aware that what they perceive as “reasonable” is not so in the eyes of the waiting patients.

### Category 2: Hospital conditions and organizational setting

The two open-ended questions in the survey elicited 704 (17.3%) statements referring to the organizational setting, uncomfortable physical conditions, lack of staffing, lack of transparency, lack of support for staff, and architectural design issues as potential contributors to the emergence of violent events.

Participants referred unequivocally to the hospital physical structure and conditions as contributing to the creation of violence. They pointed to resources, or lack thereof (workforce, physical conditions), and the manner in which the hospital administration deals with the issue of violence.

*“The conditions here are suboptimal … I, as a doctor, when I come into the ER I can’t figure out who takes care of which patient … it’s crowded and noisy…” (Resident physician)*

In addition, participants added the uncomfortable and inconvenient pathway experienced by patients upon arriving at the hospital: the security check, waiting for the triage nurse, taking of blood samples, and waiting for the physician consultation.

*“It is clear that when it is crowded, noisy, and your grandmother is lying on a gurny in the middle of the corridor, your agitation rises.” (Senior physician).*

*“The greater the pressure, the greater the violent tendency… the infrastructure isn’t designed to reduce pressure and tensions, and trying to bring about order.” (Resident physician)*

Some expressed dissatisfaction with the fact that hospital management does not act severely enough towards attackers.

*“The management has no clear policy and nobody knows what to do.” (Staff nurse)*

The contribution of the overall organizational setting to the creation of violence includes factors such as: lack of adequate and needs-based staffing leading to work overload, and poor architectural design leading to, among others, crowding of patients. While resource limits are inevitable, they are aggravated by lack of awareness of their contribution to violent occurrences and lack of a unified policy for coping with such negative effects. Additionally, survey responses indicate that 90% of the respondents had never participated in any kind of workshop regarding violence, and 40% did not even know whether there is a ward protocol for violence prevention or management.

### Category 3. Staff behavior and perceptions

The opinion of physicians about violent episodes was clear and forthright. Most staff members spoke of zero tolerance and of the need to remove the violent perpetrator immediately from the ward. As will be shown later, this does not indicate a lack of empathy but, rather, that in heat of the moment they describe a lack of willingness to seek to understand the violent behavior and its causes.

Participants stated that the characteristics of the physicians and nurses are of critical importance in coping with and preventing violence: the physician’s personality, experience, seniority, and interaction manner with nurses contribute to the probability of a violent event.

*“Look at me … I have a good relationship with nurses …. I’m a physically “big guy”… I’m senior… when a nurse tells me the patient begins to react violently, I turn to the patient, explain in an authoritative voice that the matter is closed. And it works!” (Physician)*

Head-nurses and staff nurses also related to staff conduct, especially in terms of giving explanations, guidance, and information about the system to patients. All agreed that the staff bears some of the responsibility for whether violence occurs or not.

*“I think that the attitude of some of the staff is also as problematic as the population’s is. The staffs aren’t coming to the situation with a care-giving orientation. …” (Staff nurse)*

*“We, the staff, bring about a kind of violent reaction in that we don’t behave professionally… and don’t relate in a non-condescending manner..” (Head-nurse)*

Most nurses even stated that they “work on the verge of violence all the time.”

Similarly, security personnel emphasized the contribution of the behavior of physicians and nurses to the development of a violent episode, especially verbal interaction and delayed response time. However, they also indicated that

*“It is not possible to expect or demand of physicians and nurses who work hard for long hours to behave differently.” (Security Guard)*

Besides the understanding of a possible reason for the attitude of the clinical staff, security personnel also related to their being summoned as a factor contributing to the development of a violent episode. They indicated that calling them during a violent event can escalate the situation and should be avoided if not absolutely necessary.

Supporting these findings, responses to two 4-point-scale (from minimally to greatly), survey questions regarding the occurrence of the most severe event experienced, indicated that 221 respondents (35%) reported that *their own individual behavior* contributed to some extent to the violent episode. Also, 48% reported that staff behavior contributed to creation of the event. Other factors that were perceived as contributing to the creation of the most severe violent episode experienced were patient dissatisfaction with treatment or staff attitude (45% of responses), waiting time (33% of the responses), and a comment or request made by staff that the patient did not like (20% of the responses). Responses do not sum to 100% because respondents could list more than one answer.

Qualitative data indicates that in most of the descriptions of violent episodes, nobody was managing the event which escalated and unfolded until the arrival of security staff, who separated the attacker from the attacked. Security staff felt that they were unprepared to play the role of the “responsible for managing violence”. These professionals stated they work according to guidelines (standards), but regarding violent events, they lack guidelines for action.

Thus, due to “unmanaged” violent episodes, staff asserted that their own behavior contributed to the unfolding of violence in a number of ways. Responses to the two open-ended questions in the survey produced 4047 statements, 1576 (39%) of which described staff behaviors that contributed to emergence of violence such as exhibiting a dismissive attitude, arrogance, displaying superiority, disrespect, condescension and disdain, using a blunt or a high tone of voice, extreme and violent reactions, brusqueness. (Table 3).

**Table 3:** Major Categories from Responses to Survey Open Questions

Categories	Total Responses	Percent
1. Staff behavior Dismissive attitude, arrogance, superiority, disrespect, blunt tone, extreme and violent reactions, lack of guidance and misalignment of expectations	1576	38.9%
2. Patient behavior Culture, violent attitude, sickness, anxiety, loss, drugs/alcohol/medications, psychiatric disease;feeling of loss and fear	1076	26.5%
3. Hospital setting and organizational conditions Uncomfortable physical conditions, lack of staffing, lack of transparency, lack of support for staff support, architectural design of location	704	17.3%
4. Waiting Time Waiting time for examinations, treatments, physician consultation, and imaging	384	9.5%
5. Dealing with and Resolving a Violent Event	128	3.1%
6. Others Personal and others’ stories of violent episodes	179	4.3%
Total	4047	100%

These behaviors and conditions are triggers for escalation towards violent behavior, and provide a sort of “legitimacy” for the patient and those that accompanying him/her to engage in violent behavior towards staff. Focus-groups participants frequently volunteered (often passionately) additional detail, explanation and proposed solutions. When discussing these behaviors, participants, as will be elaborated in the discussion section, reflected on “what I did that I could have done differently”. Moreover, the responses also seem to refer to not taking action that could prevent a misalignment of expectations. Behaviors that could prevent violence include coordination of expectations, guidance, introducing oneself, and showing and behaving in a respectful manner.

### Category 4: Patient behavior and perceptions

In general, focus-groups participants distinguished between “normative patients”, i.e. those who would not be expected to have significant violent tendencies, and others who, for various reasons, such as alcohol or substance abuse, might be more likely to become violent.

Head-nurses indicated that there is also a difference between “experienced” patients who are familiar with the system, and patients in their first encounter with the hospital. While all participants objected to patient violence, they sometimes identified with “normative patients”, and even stated that they would have behaved the same way.

*“There are those who react to every little thing with violence, but that is not our target population, because those who are violent, will always be violent… and it is actually people like you and me who become overwrought, I even identify with them and it is clear that if you overturn tables, you’ll get faster care.” (Resident physician)*

*“Patients in their first time visit are much less patient. When you know a patient and have an ongoing relationship, he is more patient and forgiving.” (Head-nurse)*

These findings were echoed in the responses to the open-ended questions in the survey, wherein 1076 (28.5%) referred also to other patient attributes (such as anxiety, loss, interactions of drugs and alcohol with medications, mental health problems, and fear), that can lead to violent behavior.

While staff revealed understanding and even empathy with “normative” patients’ violent outbursts, they expressed frustration and futility with the situations in which they and the patients find themselves. This emotional turmoil makes coping even more difficult. Even for “populations with violent tendencies” the staff indicated that they “accepted” or, at least, “expected” such behavior, and regarding this group they expressed more fatigue than frustration at having to provide them care. Here, a mix of “inputs” from various sources lead to violence and no one factor, such as waiting time, is solely, or even mainly responsible for the occurrence of violence in the hospital.

### Category 5: Perceptions and emotions reported by both sides

Recurring motifs regarding perceptions and emotions were found in the narratives of both sides of a violent episode: both perpetrator and staff felt attacked in turn.

### Communication

Consistently, each side involved in the violent event complained about the other side’s communicating style. Not one interviewee took responsibility for his/her own behavior. Each side focused on his/her own righteousness and made an effort to prove it.

*“If she only would have spoken to me differently.” (Family member).*

*“I will not have anyone speak to me that way.” (Staff nurse).*

### Support versus isolation

Patients were frustrated over the lack of responsiveness – “What, after all, did I request, was I asking too much?” Perpetrators reported feeling isolated, seeking support from other patients and families. Patients felt dependent on staff and feared retribution during treatment.

Differently from patients, staff expressed feeling supported by the rest of the staff. Survey answers indicate that 80% of the respondents agreed that when violence was aimed at them, they felt the support of other staff.

### Frustration

Exasperation was seen by both sides as fueling the violent event. Staff expressed frustration at being treated inappropriately - “I don’t deserve this”, and at that they did not know how to respond to violence. Moreover, clinical staff expressed escalating disappointment in the performance of the security staff when the latter did not remove the perpetrator but rather sought to reason with him and calm him down. “I don’t care about anything, when a patient becomes violent, I want that the security guards to take him out immediately. This is not my job.” Patients felt frustrated at being in such a situation, failing to obtain what they felt they needed, and not understanding “the system’s” operation and its expectations towards patients.

### Fear

Both sides felt threatened, and acted in a manner intended to demonstrate determination to the other side. A family member stated:

*“I was so afraid, frightened, when the nurse called the security personnel, but I wouldn’t let her see my fear.” (Family member)*

A staff nurse commented:

*“I was really scared. You know, you are standing in front of a big guy, I didn’t know what to do.” (Staff nurse)*

### Pressure and overload

Staff reported that they are always overloaded, and that the complex system in which they work often leaves them totally helpless. For patients, emotional strain caused by sickness, uncertainty, difficulty in coping with the disease, and the entrance to the hospital, cause feelings of intense pressure. The analysis of specificevents revealed gaps in patient and staff perceptions and expectations. A common example was a trivial request: the patient asking for his drugs before the general distribution hour, so he could go out to smoke. In his eyes, this is a legitimate request, even a right. On the other hand, the nurse was busy with other tasks and could not free herself to give the patient his drugs at that moment. The nurse felt that her acting according to ward priorities in conditions of pressure and overload was justified. As the patient pondered:

*“What’s all the noise about, what’s not legitimate in what I requested?”*

### Loss of control

All the interviewees, perpetrators and staff, evinced the strong feeling that violence occurred because “things have gotten out of control.” During the interview process itself, anger and frustration regarding the violent event seemed to ebb, hinting, perhaps, that listening and awareness can return a sense of control to all parties involved.

### Category 6. Professional roles

Differences in professional roles regarding the phenomenon of violence in the hospital were found among different types of staff. Physicians perceived the nurses as those who can prevent, or contribute, to creation of violence, and therefore, expected nurses to serve as “gate-keepers” protecting them. Physicians displayed the feeling that nurses should shield them, but they felt nurses leave them alone “at the frontline.” Nurses, on the other hand, stated that protecting physicians from violence “is not my job”. Emergency ward nurses were aware of this expectation and expressed dissatisfaction with it.

Medical staff, often try to calm the violent person themselves, and are frustrated at seeing the guards not taking a different action from the one that hasn’t worked for them. It seems there are not clear lines of coordination between medical staff and security personnel. The uncertainty created by the lack of clearly defined responsibilities and policy is played out in the roles taken by physicians and nurses during a violent event.

Figure 1 summarizes the qualitative components findings, and presents graphically the array of factors contributing to the formation of violent events. These are supported by the frequency of their mention in response to open-ended questions included in the survey, as represented in the size of each category and sub-category.

These categories enable a better understanding of the elements that constitute inputs into the creation of a violent episode. From Fig. 1, three main factors contribute, interactively, to the creation of violence in the hospital: staff behavior, patient behavior and organizational conditions. Perhaps the most interesting, but also complex and not fully understood finding is that waiting time is a black box that needs to be unpacked. It is a factor related to occurrence of violence that does not operate independently from other factors. It would appear that staff behavior can be perceived as contributing to the length of waiting time, and anger and violence directed toward staff may be reasonably thought to be linked to perceptions of patients that part of undue waiting is caused by the staff.

## Discussion

While the subject of hospital violence has been studied, the literature has fallen short by not capturing the constellation of interacting factors that contribute to violent events. These include the staff, patients and the organizational setting. The analysis of these factors lead to concrete hints that may reduce the likelihood of the eruption of violent events.

Medical staff can do much to prevent violence, and often see themselves as doing the opposite. Staff behavior can be divided into those behaviors that mitigate the creation of violence and those that contribute to its emergence. *Responsible* behaviors that could prevent violence include coordination of expectations, guidance, introducing oneself, and a respectful manner. These are the behaviors that emerged in the findings, as mentioned above, as “I should have behaved that way, but I didn’t”. On the other hand, behaviors that actively *contribute to* violence include: a brusque manner, high tone of voice, condescension and disdain. These behaviors are triggers for escalation towards violent behavior and provide a kind of “legitimacy” for the patient and those that are accompanying him/her to engage in violence towards staff. These are the behaviors that emerged in the findings evolving from might be paraphrased as “what I did that I could have done differently.”

Hospital staff are aware that they can improve their coping behaviors, providing better responses than calling upon security personnel to intervene. Tension between professions can create an unsafe environment and be conducive to violence, and placing responsibility on the shoulders of nurses makes the latter more vulnerable. Staff articulated a zero tolerance approach regarding violence aimed at them. Thus, the challenge is to combine an unequivocal demand for zero tolerance towards violence in the hospital setting, with empathy for patients, improved communication, and coordination of expectations. The reference to “things I could have done differently” may reflect staff’s wish to abide by these norms. However, there are concrete steps that need to be taken to help realize this existential desire. Many staff are unaware of guidelines dealing with prevention of violence. This, in turn, feeds-back to staff contribution to emergence of violence as staff is not prepared to deal with the phenomenon.

Little or no effort has been made by hospital management to address the need for the self-perceived lack of coping tools with violence. Management could also help reduce violent episodes by facilitating interventions aimed at improving inter-professional respect, support, and coordination.

The responsibility of patients and their relatives for their own behavior is not to be dismissed. Due to the changing role of patients in the health care system [30], the literature deals extensively with patient empowerment, generally relating to providing information, so patients can take better health-related decisions [31]. A less discussed aspect of patient empowerment is the sharing of responsibility by patients for those processes and outcomes of their own care regarding which they could legitimately be seen to influence. This responsibility-taking demand from the patient might be a double-edge sword: in an era of austerity and growing neo-liberalism in health systems, in which more and more responsibilities are “thrown” into the patients’ field, this request might manifest itself through violence [32]. Unlike this situation, what we propose here is to uncover what would be culturally sound for each health system regarding what Schutz [33] would have called moving from “the man on the street” knowledge to “the well informed citizen” regarding the expected “scripts” once entering a hospital. The passage from one category to the other could probably be facilitated by hospitals teams, “the experts”, who can walk with hospital users by, for example, providing expected pathways of the hospitalization process, including a realistic ad-hoc assessment of the situation, which was found to be effective in highly pressured contexts [34]. One such aspect is waiting time which impacts on patients, staff, and the organizational setting of violent events. Waiting times has been identified previously as a causal factor of violence [19]; the findings is this study showed that waiting times can be part of a constellation of multiple factors that contribute to the eruption of violent incidents along with staff behavior, patient behavior, hospital conditions, undefined roles and responsibilities.

The care of patients in hospitals creates situations perceived as “impossible” for all the parties involved, leading to violent episodes. Staff and patients, each from their own perspective and expectations, share conditions of overload, pressure, fatigue, and frustration, in which they also lack coping tools to prevent violence. Previous findings by this research team have demonstrated that violence takes different forms in different hospital contexts [24]. Combined with the deeper understanding of actors’ perceptions emerging from this paper, it becomes clear that in the pressured and complex hospital environment, it is inappropriate to take a linear, one-dimensional causal approach to prevention of and coping with violent episodes.

### Study limitations and directions for future research

The generalizability of single case studies is often questioned. While this study has elucidated categories of factors that are at play in episodes of hospital violence, parallel studies need to be conducted in hospitals with characteristics similar to the one described in this study. While no two cases will be produce identical results, studies should aim to capture the richness of data from both the quantitative and the qualitative components presented here. This would include the patient, staff and security personnel perspectives, together with the high response rates achieved, provide a broad, in-depth picture of the violence in hospitals. Perhaps the most prominent direction for future research involves waiting times. The findings reported only begin to untangle the interactive effects of staff behavior and waiting times and more research is required. Future research also needs to include violent episodes that do not involve security personnel (mainly due to such events being “milder”) that may remain unreported, but probably contribute to the overall atmosphere of hospitals.

## Conclusion and policy recommendations

While firmly opposing violence of any kind, health-system leaders need to examine the care-giving setting, and to develop and teach, both at the undergraduate level and in the workplace, violence mitigating tools. These include a service-oriented attitude, expectations matching and guidance, emphasis on personal responsibility (both of staff and hospital users), and awareness of the potential contribution of staff to violence, and practical scripts for preventing and dealing with violent events. The exploration of how violent events evolve, including the role of staff, is not aimed at re-assigning responsibility but rather at prevention and better coping with a growing problem that cannot be ignored in the context of hospitals.
